# The Integrin Antagonist Cilengitide Activates αVβ3, Disrupts VE-Cadherin Localization at Cell Junctions and Enhances Permeability in Endothelial Cells

**DOI:** 10.1371/journal.pone.0004449

**Published:** 2009-02-12

**Authors:** Gian Carlo Alghisi, Lionel Ponsonnet, Curzio Rüegg

**Affiliations:** Division of Experimental Oncology, Centre Pluridisciplinaire d'Oncologie (CePO), Faculty of Biology and Medicine, University of Lausanne, and NCCR Molecular Oncology, ISREC, Epalinges, Switzerland; University of Birmingham, United Kingdom

## Abstract

Cilengitide is a high-affinity cyclic pentapeptdic αV integrin antagonist previously reported to suppress angiogenesis by inducing anoikis of endothelial cells adhering through αVβ3/αVβ5 integrins. Angiogenic endothelial cells express multiple integrins, in particular those of the β1 family, and little is known on the effect of cilengitide on endothelial cells expressing αVβ3 but adhering through β1 integrins. Through morphological, biochemical, pharmacological and functional approaches we investigated the effect of cilengitide on αVβ3-expressing human umbilical vein endothelial cells (HUVEC) cultured on the β1 ligands fibronectin and collagen I. We show that cilengitide activated cell surface αVβ3, stimulated phosphorylation of FAK (Y^397^ and Y^576/577^), Src (S^418^) and VE-cadherin (Y^658^ and Y^731^), redistributed αVβ3 at the cell periphery, caused disappearance of VE-cadherin from cellular junctions, increased the permeability of HUVEC monolayers and detached HUVEC adhering on low-density β1 integrin ligands. Pharmacological inhibition of Src kinase activity fully prevented cilengitide-induced phosphorylation of Src, FAK and VE-cadherin, and redistribution of αVβ3 and VE-cadherin and partially prevented increased permeability, but did not prevent HUVEC detachment from low-density matrices. Taken together, these observations reveal a previously unreported effect of cilengitide on endothelial cells namely its ability to elicit signaling events disrupting VE-cadherin localization at cellular contacts and to increase endothelial monolayer permeability. These effects are potentially relevant to the clinical use of cilengitide as anticancer agent.

## Introduction

Endothelial cell - matrix interactions mediated by integrin adhesion receptors play a critical role in vascular development, angiogenesis and vascular homeostasis [1]. Integrins are heterodimeric cell surface complexes formed by non-covalently associated α and β subunits, consisting of large extracellular domains, single transmembrane spanning domains and short cytoplasmic tails. A particular feature of integrins is their tight regulation of ligand binding activity. Transition from a low to a high affinity state (“affinity maturation”) can be induced by intracellular signaling events (“inside-out” signaling) or by high-affinity ligands [2]. Ligand binding induces allosteric changes in the receptor conformation, leading to the activation of intracellular signaling pathways, including the Ras-MAPK, PI3K-PKB-mTOR and small GTPases (e.g. Rho, Rac) pathways (“outside-in” signaling) [2]. Since integrins do not possess intrinsic enzymatic activities they require interaction with cytoplasmic adaptor molecules and kinases, including FAK and Src-family kinases, to transduce signaling events. Integrin-mediated signaling is critical for the stabilization of cell adhesion and the promotion of cell migration, proliferation and survival [2].

Integrin αVβ3 is expressed at low levels on quiescent endothelial cells, while it is strongly induced on angiogenic endothelial cells present in granulation tissue and cancer, and is considered as an attractive therapeutic target to inhibit pathological angiogenesis [3]. Pharmacological inhibition of αVβ3 suppresses angiogenesis in many experimental models and αVβ3 antagonists (i.e. antibodies, peptides and peptidomimetics) are being developed as antiangiogenic drugs [4]. Cilengitide [5] (EMD121974) is a cyclic Arg-Gly-Asp (RGD)-derived peptide binding with high affinity to αVβ3 (IC_50_ of 0.6 nM) and inhibiting αVβ3 and αVβ5-dependent adhesion [6]. Cilengitide displays antiangiogenic effects *in vitro* [7] and *in vivo* [8]–[10]. It exerts antitumor effects against experimental melanoma and brain tumors [8], [9], [11], [12], it sensitizes endothelial cells to TNF cytotoxicity *in vitro* [13] and enhances antitumor effects of chemotherapy [14] and radiotherapy [15] *in vivo*. Cilengitide is in clinical development as anticancer drug. As a single agent it is well-tolerated [16] and shows evidence of durable responses in patients with recurrent gliomas [17], [18]. In combination with chemotherapy it showed evidence of activity in pancreas cancers [19] and in highly vascularized head and neck tumors [20]. Cilengitide is now in phase III clinical testing in glioblastoma in combination with radio- and chemotherapy [21].

It is generally assumed that the antiangiogenic activity of cilengitide is due to the inhibition of sprouting and differentiation and the induction of anoikis of angiogenic endothelial cells relaying on αVβ3/αVβ5 for adhesion and survival [7], [22]. However, in addition to αV integrins, angiogenic endothelial cells express multiple integrins, including α1β1, α2β1, α4β1, α5β1, α6β1, and α6β4, which are not targeted by cilengitide [3]. Adhesion through these integrins might compromise the antiangiogenic activity of cilengitide. Little is known on the effect of cilengitide on endothelial cells expressing αVβ3/αVβ5 but adhering mostly through other integrins, in particular those of the β1 family.

To address this question, we examined the effect of cilengitide on HUVEC, which express αVβ3, under condition of β1 integrin-mediated adhesion. Here we demonstrate that HUVEC exposure to cilengitide results in the phosphorylation of Src, FAK and VE-cadherin, the accumulation of αVβ3 at the cell edge, the disappearance of VE-cadherin from cell-cell contacts and the increase in HUVEC monolayer permeability.

## Results

### Cilengitide causes disappearance of αVβ3 from focal adhesions and promotes its accumulation at the cell periphery

**Figure 1:**
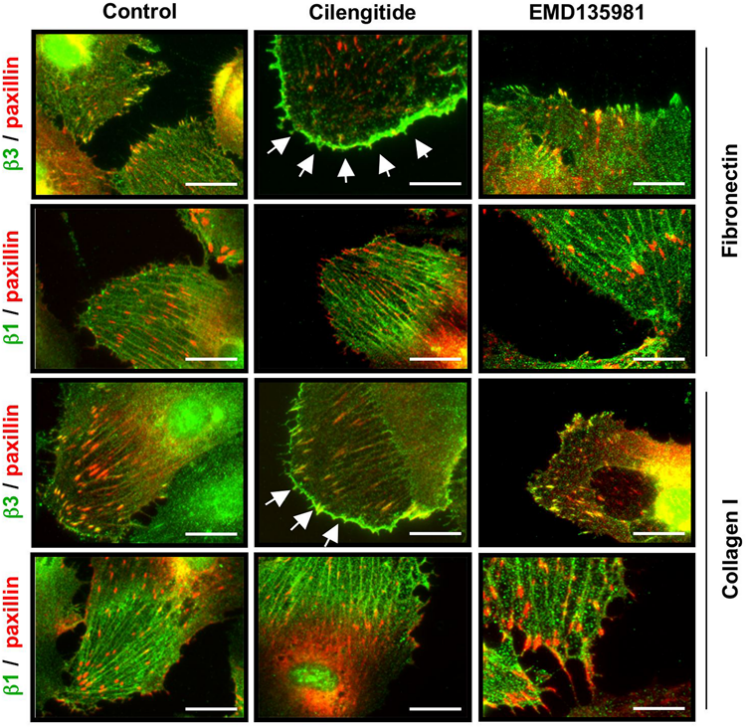
Cilengitide causes loss of αVβ3 from focal adhesions and promotes appearance of αVβ3 patches at the cell edge. HUVEC were plated on coverslips coated with fibronectin or collagen I and were treated with 10 µM of cilengitide for 20 minutes. The localization of the αVβ3 or β1 integrin (green) and paxillin (red) were monitored by immunofluorescence staining. In HUVEC plated on fibronectin αVβ3 was present at focal adhesions, while β1 was present at fibrillar adhesions. Cilengitide, but not EMD 135981, caused loss of αVβ3 from focal adhesions and appearance of αVβ3-positive thin patches at the cell edge (arrows). β1 localization was not altered by cilengitide. A similar effect on αVβ3 (arrows) was observed on cells plated on collagen I, with the difference that focal adhesions were less abundant on this matrix. Optical magnification: 400×; Bar: 10 µm. (n = 5).

Cilengitide efficiently inhibits αVβ3-mediated cell adhesion and induces detachment of endothelial cells cultured on αVβ3 ligands, such as vitronectin or gelatin [13]. To test the effect of cilengitide on endothelial cells expressing αVβ3 but mostly using β1 integrins for their adhesion, we seeded HUVEC on fibronectin and collagen I. HUVEC use predominantly α5β1 to adhere to fibronectin (with minor contribution of αVβ3) and α1β1/α2β1 to adhere to collagen I [23]. Subsequently we exposed adherent HUVEC to cilengitide at a clinically-relevant concentration (i.e. 10 µM, [17]) or EMD135981, an Arg-Ala-Asp (RAD)-based inactive cyclopeptide. First we monitored the effect of cilengitide on αVβ3 localization. In HUVEC plated on fibronectin, αVβ3 was present at focal adhesions while β1 integrins clustered at fibrillar adhesions, as previously observed [24]. Cilengitide, but not EMD135981, caused loss of αVβ3 from paxillin-positive focal adhesions and promoted the appearance of thin, αVβ3-positive and paxillin negative linings at the cell edge (Figure 1, arrows). The localization of β1 integrins at fibrillar adhesions was not perturbed by cilengitide. HUVEC cultured on collagen I showed fewer focal adhesions while had well-developed fibrillar adhesions. Cilengitide treatment induced αVβ3 accumulation at the cell border without affecting β1 integrin localization (Figure 1).

### Cilengitide causes VE-cadherin disappearance from cellular junctions

**Figure 2:**
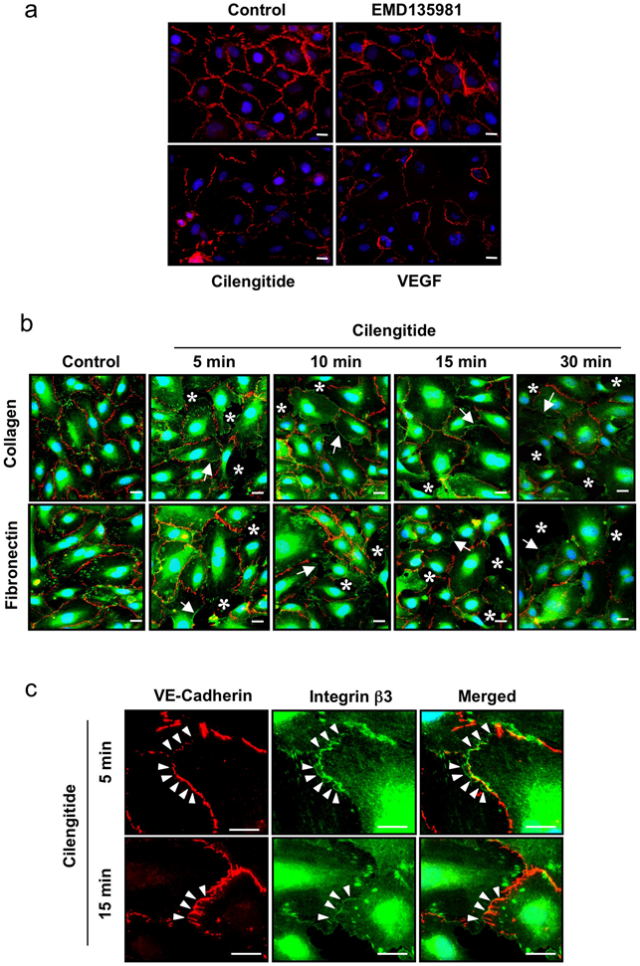
Cilengitide induces VE-cadherin loss form cellular junctions. (a) Confluent HUVEC plated on fibronectin, were exposed to cilengitide or EMD135981 (10 µM each) or VEGF (100 ng/ml) for 20 minutes and stained for VE-cadherin. Cilengitide and VEGF treatments disrupted VE-cadherin localization at cellular junctions, while EMD135981 showed no effect (n = 3). Optical magnification: 400×; Bar: 10 µm. (b) Confluent HUVEC plated on fibronectin or collagen I were exposed to cilengitide (10 µM each) for the indicated time and double stained for VE-cadherin and β3 integrin. Cilengitide disrupted VE-cadherin staining and promoted appearance of β3 at VE-cadherin-depleted cell-cell borders (arrows). Paralleling loss of VE-cadherin from cell-cell junctions, ‘gaps’ appeared in the monolayer (asterisks). (n = 4). Optical magnification: 400×; Bar: 10 µm. (c) Higher magnification (2× zooming in) of HUVEC cultures of the experiment shown in panel b to demonstrate rare co-localization of VE-cadherin and β3 integrin at cellular junctions upon cilengitide stimulation (arrowheads). (n = 4). Bars: 10 µm.

VE-cadherin is a major endothelial cell junctional molecule mediating cell–cell adhesion [25]. It has been previously reported that integrin ligation through multivalent fibronectin-coated beads disrupted VE-cadherin-containing adherens junctions in bovine aortic endothelial cells [26]. We therefore tested, whether αVβ3 ligation by monovalent cilengitide affected VE-cadherin localization. In confluent monolayers of HUVEC cultured for 18 hours on fibronectin or collagen I, VE-cadherin was localized at cell-cell contacts (Figure 2a and data not shown). Addition of cilengitide markedly disrupted VE-cadherin localization at cellular junctions, while the EMD135981 peptide was ineffective. Stimulation with VEGF also caused VE-cadherin disappearance from cellular junctions (Figure 2a), consistent with previous reports [27].

Next, we performed αVβ3 and VE-cadherin co-staining experiments to monitor the spatial relationship between the appearance of αVβ3 at the cell periphery and loss of VE cadherin from cell-cell junctions. In confluent HUVEC cultures VE-cadherin and αVβ3 were localized at different locations (cell-cell contacts and focal adhesions, respectively) (Figure 2b, control). Upon stimulation with cilengitide, VE-cadherin staining became discontinuous and αVβ3 appeared at cell borders, typically at sites where VE-cadherin disappeared from cellular contacts (Figure 2b, time course). Paralleling loss of VE-cadherin from cell-cell junctions, ‘gaps’ appeared in the monolayer (Figure 2b, asterisks), consistent with diminished cell-cell adhesion and cell retraction. Concomitant presence of VE-cadherin and αVβ3 at cell-cell contacts was very rarely observed (Figure 2c, arrowheads), suggesting that co-localization of αVβ3 and VE-cadherin is a rather mutually exclusive event.

Taken together, these results indicate that exposure of confluent HUVEC to cilengitide while cultured on fibronectin or collagen I, resulted in the redistribution of αVβ3 from focal adhesions to the cell periphery and the concomitant disappearance of VE-cadherin from cellular junctions.

### Cilengitide activates cell surface αVβ3 integrin

**Figure 3:**
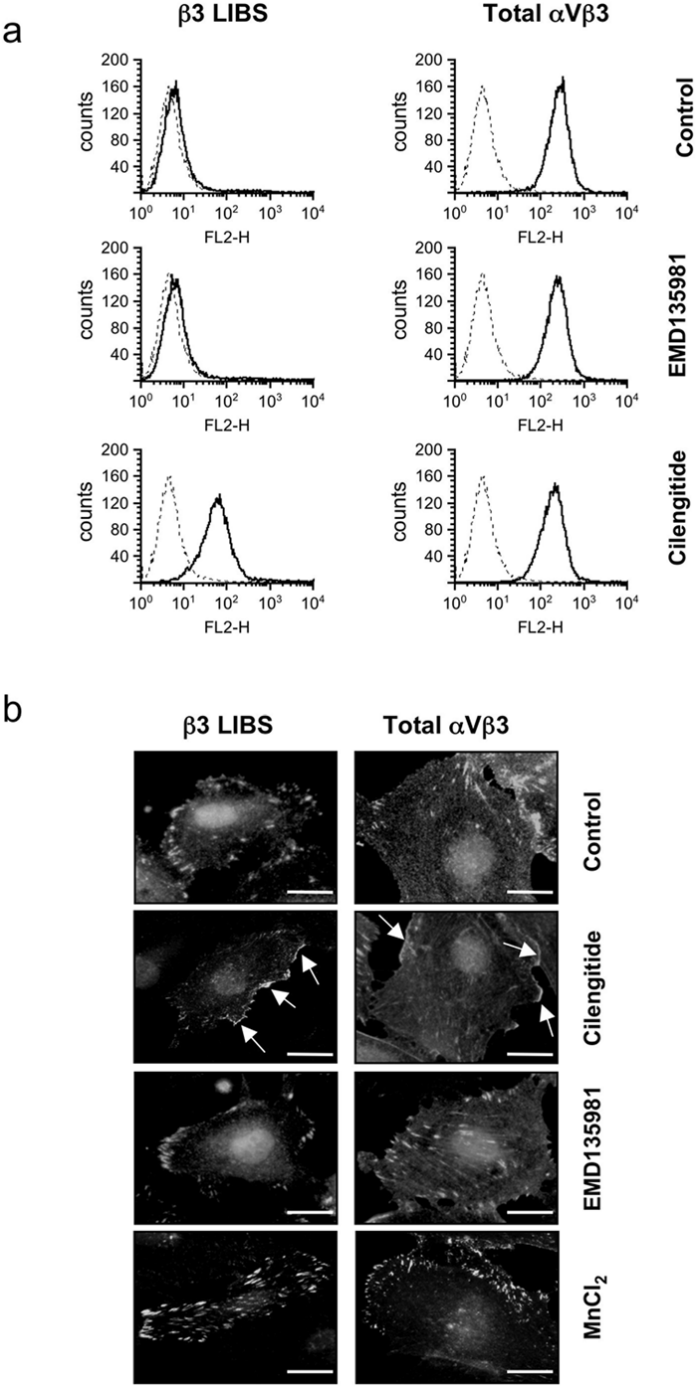
Cilengitide activates αVβ3 on HUVEC. (a) HUVEC in suspension were exposed to 10 µM of cilengitide or EMD135981 for 10 minutes, stained by immunofluorescence for β3 LIBS and total αVβ3 expression (with LIBS-1 and LM609 mAbs, respectively) and analyzed by flow cytometry. Cilengitide, but not EMD135981, induced LIBS expression (left histograms, thick lines), without affecting total αVβ3 expression (right histograms, thick lines). Dotted lines: cellular fluorescence in the absence of primary antibody. (n = 3). (b) Fibronectin-adherent HUVEC were exposed to 10 µM cilengitide, EMD135981, or 1 mM MnCl_2_ for 10 minutes, stained for β3 LIBS and total αVβ3 expression (with CRC54 or LM609 mAbs, respectively) and analyzed by immunofluorescence microscopy. Total αVβ3 and β3 LIBS were present at focal adhesions in unstimulated HUVEC and at tiny patches at the cell edge in cilengitide-exposed HUVEC, thus confirming that αVβ3-positive patches contain active αVβ3. MnCl_2_ stimulated recruitment and activation of αVβ3 at focal adhesions. (n = 2). Optical magnification: 400×; Bars: 10 µm.

The disappearance of VE-cadherin from cell-cell contacts suggested that cilengitide-bound integrin αVβ3 might initiate intracellular signaling events by activating αVβ3. To test this hypothesis we monitored the capacity of cilengitide to induce affinity maturation of αVβ3 on endothelial cells using antibodies (i.e. LIBS-1 and CRC54) recognizing ligand-induced binding sites (LIBS) on β3 integrins [28], [29]. Cilengitide, but not EMD135981, induced LIBS-1 and CRC54 epitope expression on HUVEC in suspension, without altering total cell surface levels of αVβ3 as detected by LM609 mAb (Figure 3a). Addition of MnCl_2_, a known integrin activator, also induced LIBS-1 and CRC54 epitope expression (data not shown) as previously reported [24]. Cilengitide had no effect on β1 LIBS expression as detected by mAb HUTS-21 (data not shown). To test whether cilengitide-induced affinity maturation also occurred on adherent HUVEC, we exposed fibronectin-adherent HUVEC to cilengitide, EMD135981 or MnCl_2_ and stained them with CRC54 (LIBS-1 mAb does not work on fixed cells) and LM609. In unstimulated HUVEC, focal adhesions were positive for CRC54, consistent with the ligated/active state of αVβ3 (Figure 3b). Upon cilengitide stimulation we observed CRC54-positive patches at the cell periphery, consistent with a cilengitide-ligated (activated) state (Figure 3b, arrows). In comparison, MnCl_2_ treatment enhanced αVβ3 clustering and expression of the CRC54 epitope at focal adhesions as already reported [24].

### Cilengitide induces Src and FAK phosphorylation

**Figure 4:**
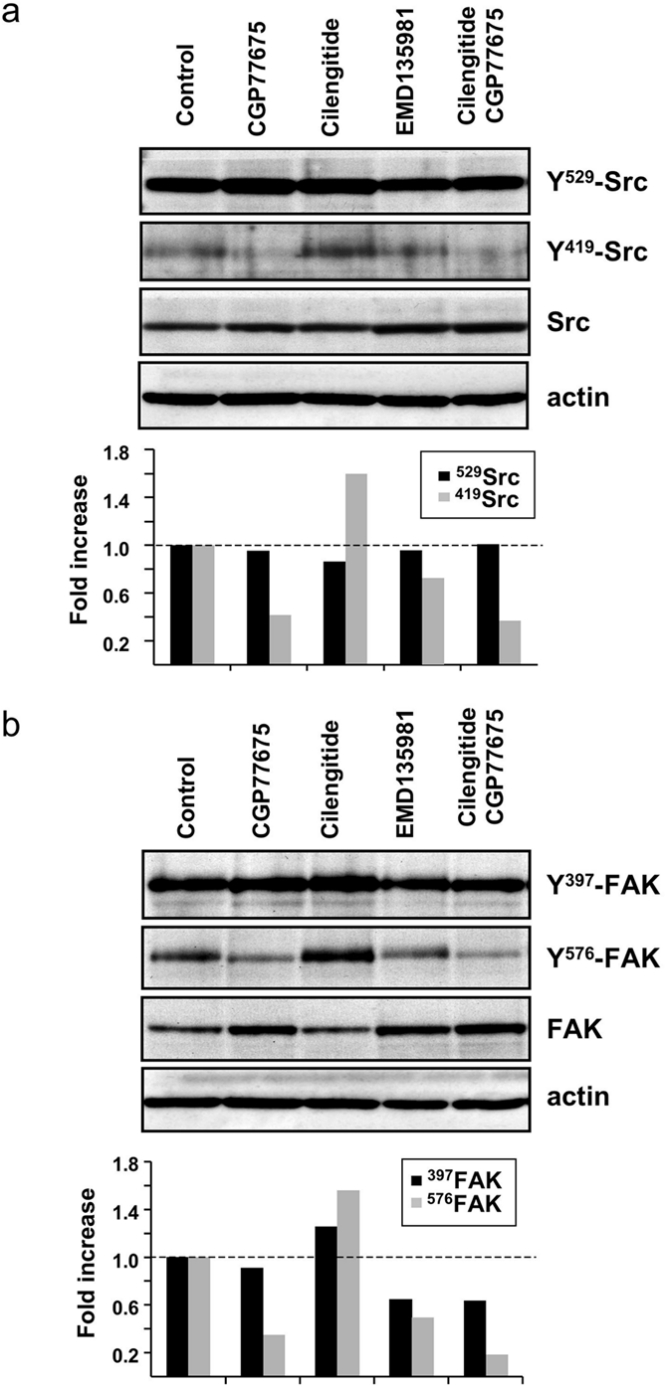
Cilengitide induces Src and FAK phosphorylation. (a) Western blotting analysis of Src phosphorylation at Y^529^ and Y^419^ and total Src in HUVEC grown on fibronectin and exposed for 10 minutes to EMD135981, cilengitide (10 µM each) and CGP77675 (2.5 µM) as indicated. Cilengitide increased Src phosphorylation at Y^419^ but did not alter Y^529^ phosphorylation. CGP77675 prevented Y^419^ phosphorylation. (b) Western blotting analysis of the same cells as in panel a, but for phosphorylation of FAK at Y^397^ and Y^576^ and total FAK. Cilengitide increased FAK phosphorylation at both tyrosine residues and this was inhibited by CGP77675. EMD135981 had no effect on Src or FAK phosphorylation. Actin was detected to demonstrate equal loading of the lanes. The bar graph represents the relative level of phospho Src/FAK over total Src/FAK as determined by band density analysis. (n = 3).

Next we sought after evidence for cilengitide-induced intracellular signaling events. Src-dependent phosphorylation of focal adhesion kinase (FAK) is one of the first signaling events initiated by integrin activation [2], [3]. Src, like other Src family kinases, is negatively regulated though the phosphorylation of a carboxyl-terminal tyrosine residue (Y^529^ in human Src). This phosphorylation forces the Src C-terminal domain to interact with the SH2 and SH3 domains, thus forming a loop that masks the Src kinase domain [30]. Disruption of this loop, achieved through protein tyrosine phosphatases (i.e. PTPα, PTPIB, Shp2) –mediated dephosphorylation of Y^529^, or via integrin clustering in the absence of Y^529^ dephosphorylation [31], allows Src to interact with its substrates via SH2 and SH3 domains. Cilengitide treatment of confluent HUVEC, increased Src phosphorylation of tyrosine residue Y^419^ without decreasing phosphorylation of Y^529^ (Figure 4a), consistent with integrin-mediated Src activation, and promoted FAK phosphorylation at tyrosine residues Y^576^ and Y^577^, two well-characterized phosphoacceptor sites of Src [32], relative to non-treated or EMD135981-treated cells (Figure 4b). The Src kinase inhibitor CGP77675 [33] suppressed Y^419^ Src and Y^576^ FAK phosphorylation (Figure 4a and 4b).

### Cilengitide induces VE-cadherin phosphorylation at residues Y^658^ and Y^731^

**Figure 5:**
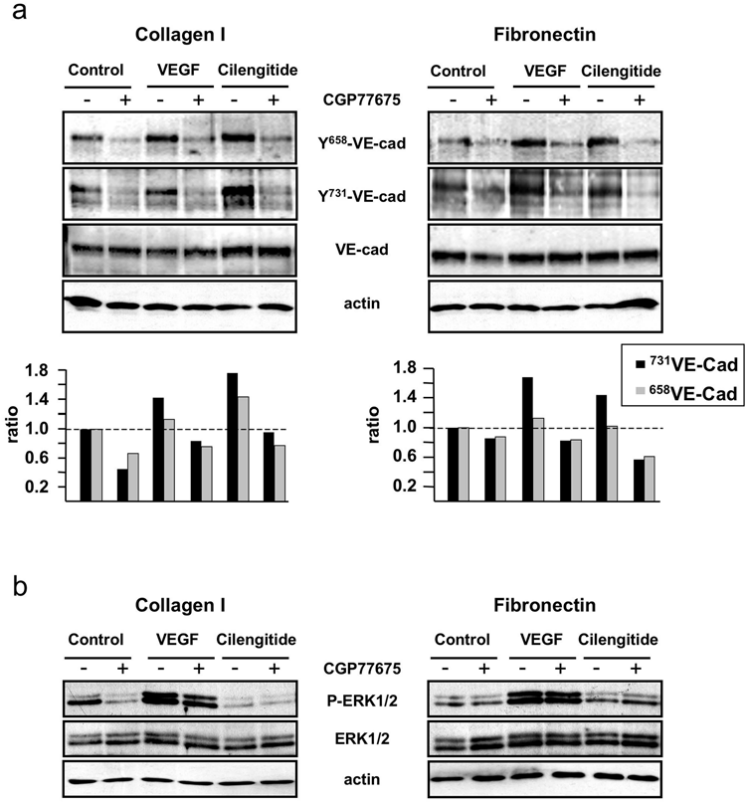
Cilengitide induces Src-dependent phosphorylation of VE-cadherin cytoplasmic domain. (a) Western blotting analysis of VE-cadherin phosphorylation at tyrosine residues Y^658^ and Y^731^ and total VE-cadherin in HUVEC grown on fibronectin or collagen I and stimulated for 10 minutes with cilengitide (10 µM) or VEGF (100 ng/ml) in the presence or absence of CGP77675. Cilengitide treatment increased VE-cadherin phosphorylation at Y^658^ and Y^731^, while VEGF enhanced Y^658^ phosphorylation only. The bar graph represents the relative level of phospho VE-cadherin over total VE-cadherin as determined by band density analysis. (b) Western blotting analysis of phospho and total ERK 1/2 of the same cultures as in panel a. VEGF activated ERK 1/2, while cilengitide did not. Actin was detected to demonstrate equal loading of the lanes. (n = 3).

**Figure 6:**
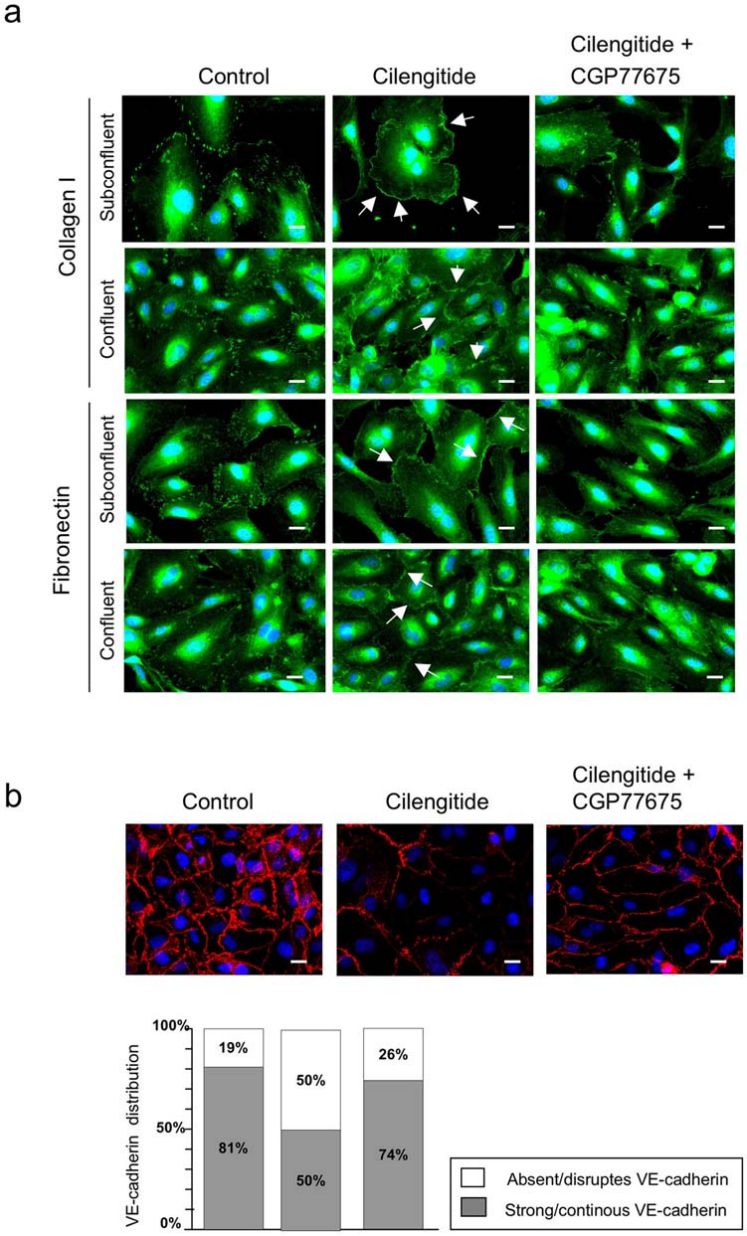
Src inhibition prevents cilengitide-induced relocalization of αVβ3 at the cell edge and disappearance of VE-cadherin from cellular junctions. (a) Subconfluent and confluent HUVEC cultured on fibronectin or collagen I were exposed for 20 minutes to cilengitide (10 µM) in the presence of absence of CGP77675 (2.5 µM) and stained for αVβ3. Cilengitide-induced recruitment of αVβ3 to the cell edge (arrows) and this was prevented by CGP77675. (b) Confluent HUVEC cultured on fibronectin were treated for 20 minutes with cilengitide in the presence or absence of CGP77675. CGP77675 prevented cilengitide-induced VE-cadherin loss from cell-cell contacts. The bar graph gives the quantification of VE-cadherin staining at cell borders. The white and gray segments of the bars represent absent/disrupted *vs.* strong/continuous VE-cadherin staining, respectively (see material and methods for details). (n = 3). Optical magnification: 400×; Bars: 10 µM.

Src was shown to phosphorylate the VE-cadherin cytoplasmic domain in response to VEGF stimulation [34]. We therefore tested whether cilengitide induced Src activation resulted in the phosphorylation of the VE-cadherin cytoplasmic domain. Cilengitide treatment induced VE-cadherin phosphorylation at Y^658^ and Y^731^, which correspond to the binding sites for p120 catenin and β-catenin, respectively [35]. Addition of CGP77675 (2.5 µM) strongly reduced basal and cilengitide-induced phosphorylation of both residues (Figure 5a). As reported, VEGF stimulation induced Y^658^ phosphorylation and to a lesser extent Y^731^ phosphorylation, which were also inhibited by CGP77675. In contrast to VEGF, however, cilengitide did not induce phosphorylation of MEK 1/2, Akt, and Iκ-B, (Figure 5b and data not shown). Next we tested the effect of inhibition of Src kinase activity on the recruitment of αVβ3 to the cell periphery and the disappearance of VE-cadherin from cell junctions. Indeed, CGP77675 prevented the formation of αVβ3 patches at the cell edge in HUVEC plated on fibronectin or collagen I at both sub-confluent and confluent conditions, and attenuated the disappearance of VE-cadherin from cell-cell contacts induced by cilengitide (Figure 6).

Taken together these results establish that cilengitide induces αVβ3 affinity maturation, and initiates signaling events in endothelial cells leading to phosphorylation of Src, FAK and VE-cadherin. These phosphorylation events, recruitment of αVβ3 at the cell periphery and disappearance of VE-cadherin from cellular contacts requires Src kinase activity.

### Cilengitide enhances HUVEC monolayer permeability

**Figure 7:**
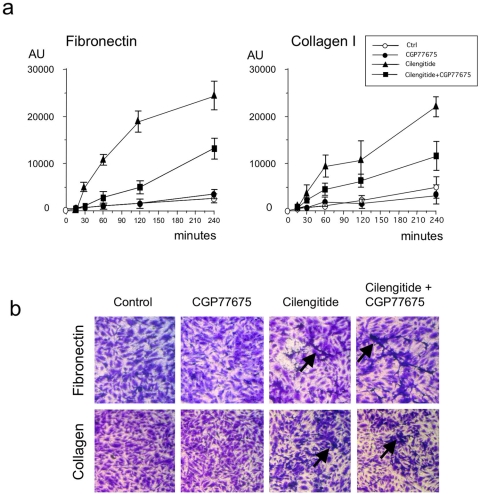
Cilengitide augments the permeability of HUVEC monolayers. (a). HUVEC were grown on fibronectin- or collagen I-coated PET filter inserts for 20 hours to ensure confluence and treated with cilengitide (10 µM), CGP77675 (2.5 µM) or a combination thereof. Permeability was measured using the tracer molecule FITC-dextran. Cilengitide increased HUVEC monolayer permeability on both matrices and CGP77675 only partially prevented this increase. Results represent the increase in permeability of treated cultures relative to untreated controls at t = 0 and is given in arbitrary fluorescence units (AU). (b) Crystal violet staining of control and treated filters at the end of the assay (240 minutes) revealed that cilengitide did not cause extensive detachment of HUVEC but induced the appearance of retraced, dendritic-like cells (arrows). (Triplicate filters/condition, n = 3).

VE-cadherin-mediated cell-cell adhesion and integrin-mediated cell-matrix adhesion are essential for maintaining endothelial cell monolayer tightness [36], [37]. Based on the above observations, we set up to test whether cilengitide treatment increased permeability of confluent HUVEC. Addition of cilengitide (10 µM) to HUVEC cultured on fibronectin or collagen-coated filter inserts, resulted in a time-dependent increase in transendothelial permeability (Figure 7a). Microscopic examination of the filters at the end of the assay (240 minutes) revealed that cilengitide induced morphological changes to the cultures, in particular the appearance of dark (dense) dendritic-like cells, consistent with cells that retracted or detached from the substrate (Figure 7b, arrows). CGP77675 (2.5 µM) partially abolished cilengitide-induced increased permeability but was ineffective in preventing the appearance of retracted cells (Figure 7a and 7b). As expected treatment of HUVEC cultured on vitronectin-coated filters resulted in massive cell detachment and increased permeability, consistent with αVβ3/αVβ5-mediated adhesion to this substrate (data not shown).

### Cilengitide interferes with β1 integrin-dependent HUVEC attachment on low-density ligands

**Figure 8:**
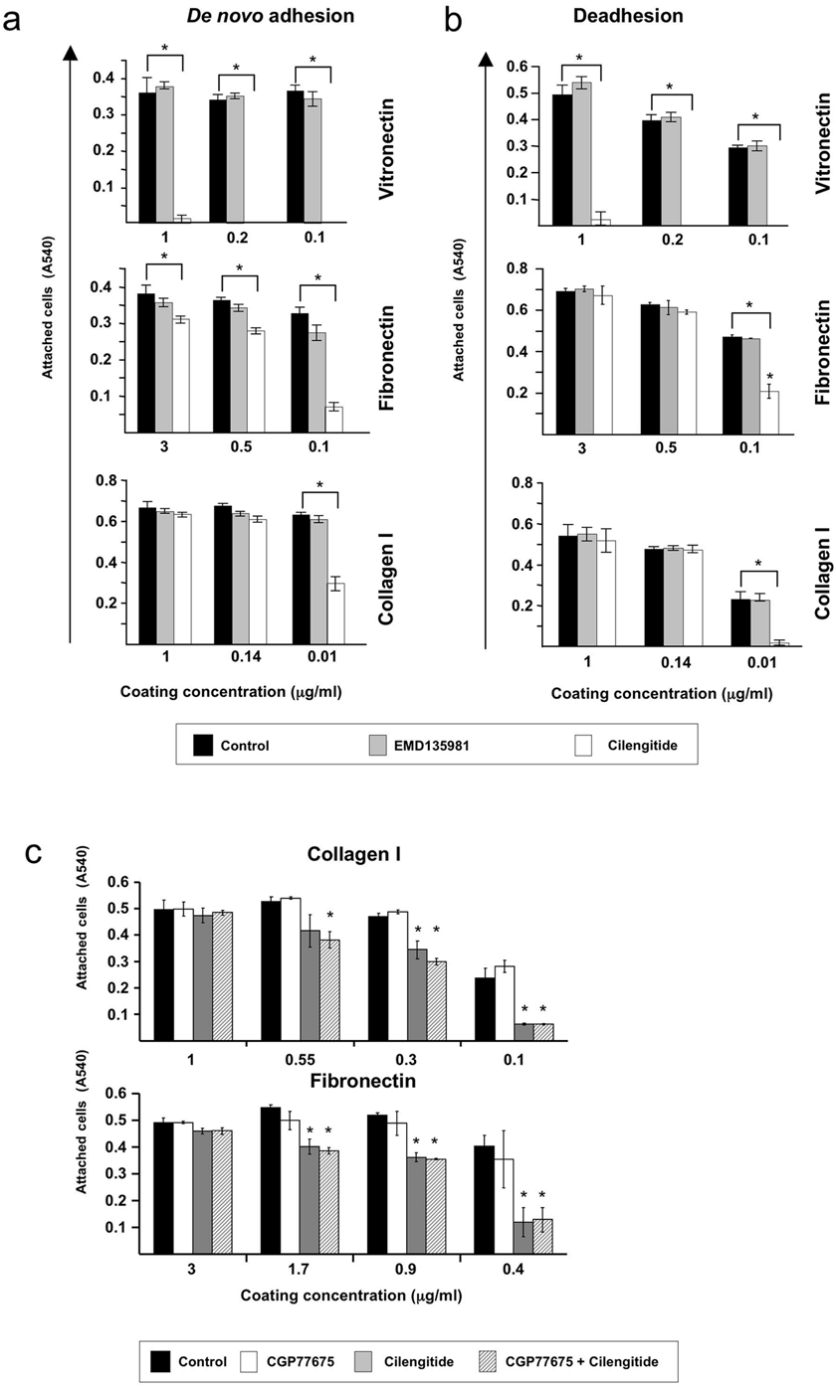
Cilengitide interferes with HUVEC adhesion on low-density β1 integrin substrates. (a) HUVEC short-term adhesion assays performed on vitronectin (αVβ3 ligand), fibronectin (α5β1>αVβ3 ligand) and collagen I (α1β1/α2β1 ligand) coated at the indicated concentrations, in medium only (black bars) or in the presence of EMD135981 (gray bars) or cilengitide (white bars). On vitronectin, cilengitide inhibited adhesion at all coating concentrations while on fibronectin and collagen I it blocked adhesion only at low coating concentrations. (n = 5). (b) HUVEC detachment assays. HUVEC were cultured for 18 hours on vitronectin, fibronectin or collagen I coated at the indicated concentrations, to allow for full attachment, before exposure for 4 hours to medium only (black bars), EMD135981 (gray bars) or cilengitide (white bars). Cilengitide detached HUVEC cultured on vitronectin at all coating concentrations, while it induced HUVEC detachment from fibronectin and collagen only at low coating concentrations (Triplicate wells/condition, n = 3). (c) HUVEC short-term adhesion assays performed on fibronectin and collagen I coated at the indicated concentrations, in medium only (black bars), in the presence of CGP77675 (white bars), cilengitide (gray bars), cilengitide+CGP77675 (hatched bars). Src inhibition did not prevent cilengitide-induced inhibition of cell adhesion on low matrix concentrations. (Triplicate wells/condition, n = 2). Attached cells were quantified by Crystal Violet staining and OD determination at 540 nm wavelength. Asterisks indicate statistical significant differences of the values relative to untreated controls (p<0.05).

The appearance of retracted HUVEC in cilengitide-treated filter inserts during the permeability assays on fibronectin and collagen I, suggested the possibility that cilengitide might interfere with adhesion on fibronectin or collagen I. Activation of one individual integrin was previously shown to interfere with the function of other integrins though a transdominant negative effect [38]. To test whether cilengitide-induced αVβ3 activation might interfere with β1 integrin-mediated adhesion, we first performed short-term adhesion assays on vitronectin (as αVβ3 ligand), fibronectin (as mixed α5β1>αVβ3 ligand) or collagen I (as β1 ligand). Since the transdominant negative effect is based on the competition for intracellular adaptor and signaling molecules among unclasped cytoplasmic β tails of active integrins [39], and the stoechiometry of active (i.e. αVβ3) *vs* target (i.e. β1) integrins is critical, we tested the effect of cilengitide on HUVEC engaging decreasing levels of β1 integrins by coating decreasing concentrations of ligands. Cilengitide prevented αVβ3-mediated HUVEC adhesion to vitronectin at any coating concentrations, consistent with a direct inhibition of αVβ3 ligand binding activity (Figure 8a). Cilengitide showed no effect on β1-mediated HUVEC adhesion on fibronectin and collagen I coated at high concentrations, while it interfered with HUVEC adhesion to low ligand concentrations (Figure 8a). To test the effects of cilengitide on cells already attached, we added cilengitide to HUVEC cultured for 18 hours in wells coated with graded amounts of vitronectin, fibronectin or collagen I. Cilengitide induced detachment of HUVEC cultured on vitronectin regardless of the coating concentration, while it detached HUVEC from fibronectin and collagen I only in wells coated with low protein concentrations (Figure 8b). Addition of CGP77675 did not abolish the anti-adhesive effect of cilengitide on HUVEC plated on low-density fibronectin or collagen I in a short-term adhesion assay (Figure 8c). Sub G1 DNA content analysis of control and treated cultures revealed an increased frequency of Sub G1 DNA containing-cells in wells coated with fibronectin or collagen I at low densities and exposed to cilengitide, consistent with detachment-induced death (Table 1) [40].

**Table 1:** Relative cell death of HUVEC cultures exposed to cilengitide or EMD135981.

Matrix protein coated	Matrix protein coating concentration	Treatment	
		EMD135981	Cilengitide
Vitronectin	High (1 µg/ml)	<1%	59%
Vitronectin	Low (0.1 µg/ml)	<1%	58%
Fibronectin	High (3 µg/ml)	<1%	<1%
Fibronectin	Low (0.1 µg/ml)	<1%	16%
Collagen I	High (1 µg/ml)	<1%	<1%
Collagen I	Low (0.01 µg/ml)	7%	41%

HUVEC were cultured for 18 hours on vitronectin (αVβ3 ligand), fibronectin (α5β1>αVβ3 ligand) and collagen I (α1β1 and α2β1 ligand) before they were exposed for 4 hours to EMD135981 or cilengitide. Cells were collected and Sub-G1 DNA content determined by propidium iodide staining and flow cytometry analysis. Results are expressed as percent increase of cell death relative to untreated conditions. Cilengitide increased HUVEC cell death on vitronectin at high and low protein coating concentrations, while on fibronectin and collagen it only did it at low protein coating concentrations. (n = 3).

Taken together, these results demonstrate that cilengitide interferes with β1-mediated adhesion under conditions of limited β1-substrate concentration and limited β1 engagement. This effect is independent of Src activity and is consistent with a β3 to β1 transdominant negative effect.

## Discussion

**Figure 9:**
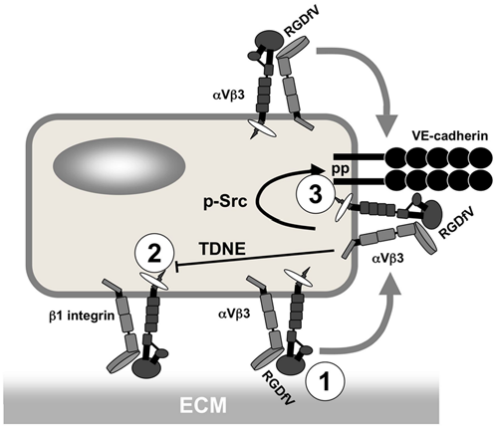
Proposed model of cilengitide effects on endothelial cells. Cilengitide acts on αVβ3-expressing endothelial cells in three ways: 1, it suppresses αVβ3-dependent adhesion by directly inhibiting αVβ3-ligand-binding function; 2, it interferes with β1 integrin-mediated cell adhesion through a transdominant negative effect induced by activated αVβ3; 3, it stimulates phosphorylation of VE-cadherin cytoplasmic domain and disrupts VE-cadherin localization at cell-cell contacts through activation of αVβ3 and Src-dependent signaling. Abbreviations: ECM, extracellular matrix; RGDfV, cilengitide; TDNE, transdominant negative effect; pp, phosphorylation.

The antiangiogenic activity of cilengitide is largely attributed to its ability to directly interfere with αVβ3/αVβ5-mediated adhesion of angiogenic endothelial cells, thereby inducing cell detachment-mediated death (anoikis) of cells relying on these integrins for adhesion and survival [5], [40]. Angiogenic endothelial cells, however, in addition to αVβ3 and αVβ5, express other integrins, in particular α1β1, α2β1, α4β1, α5β1, α6β1, and α6β4, which are not targeted by cilengitide [3]. Based on the current model of action of cilengitide, endothelial cells expressing and using these integrins would be insensitive to cilengitide effects. The present work was initiated to test whether endothelial cells expressing αVβ3, but predominantly using β1 integrins for adhesion, are insensitive to cilengitide, or whether they may indeed show some effects. Here we report five effects of cilengitide under such conditions: i) affinity maturation of αVβ3 and its accumulation to the cell periphery; ii) phosphorylation of Src (Y^419^), FAK (Y^397^ and Y^576/577^) and VE-cadherin (Y^658^ and Y^731^); iii) disappearance of VE-cadherin from cell-cell contacts; iv) detachment of HUVEC cultured on low-density β1 substrates; v) increased HUVEC monolayer permeability. These findings unravel a more complex picture of the mechanistic effects of cilengitide on endothelial cells and, more generally, highlight the role of integrins and integrin-induced signaling events in the regulation of endothelial cell functions (See Figure 9 for a working model).

There is structural evidence that high affinity RGD-based cyclopeptides, including cilengitide, can induce large-scale conformational changes of soluble truncated integrins consistent with ligand-induced activation [41], [42]. RGD-based soluble ligands were shown to induce some signaling events, such as intracellular calcium mobilization in neurons, smooth muscle cells and T lymphocytes [43], [44], or protein kinase C activation in oocytes [45]. Our work extends these findings by demonstrating that a monomeric, high-affinity RGD-based ligand induces affinity maturation (e.g. activation) of cell surface αVβ3 leading to Src, FAK and VE-cadherin phosphorylation. The mechanism by which cilengitide elicits these signaling events remain to be determined. Current knowledge implies integrin clustering induced by immobilized or soluble-multivalent ligands as an essential step to recruit adaptor proteins or kinases and initiate signaling events. Our results demonstrate that a monovalent high-affinity ligand is nevertheless sufficient to elicit some signaling events. A plausible explanation is that since Src is constitutively associated with the cytoplasmic domain of the β3 subunit, cilengitide-induced αVβ3 ‘outside-in’ activation and unclasping of the αVβ3 cytoplasmic domains is sufficient to activate Src. Active Src can then complex with FAK resulting in mutual Src-FAK phosphorylation promoting lateral association of cilengitide-occupied αVβ3 through its SH3 domain, resulting in patches formation at the cell periphery [46]. αVβ3 activation by cilengitide, however, appears insufficient to fully activate downstream signaling pathways, such as ERK1/2, NF- κB or PI3K/Akt probably due to the lack of additional adaptor and signaling proteins normally present at focal adhesions [3]. The mutually exclusive localization of αVβ3 and VE-cadherin at cellular junctions in confluent HUVEC suggests that a causal link between cilengitide-induced αVβ3 and Src activation and the disappearance of VE-cadherin from cell-cell contacts. In our model activated αVβ3 recruiting at cell-cell junctions brings active Src to VE-cadherin-catenin complexes, thereby promoting VE-cadherin phosphorylation at residues Y^658^ and Y^731^, dissociation from β- and p120 catenins and disappearance from cell-cell contacts, consistent with published results [26], [35].

The observed increased permeability induced by cilengitide is consistent with phosphorylation of VE-cadherin and disappearance from cell-cell contacts. However, in contrast to phosphorylation and displacement of VE-cadherin, which could be effectively prevented by pharmacological inhibition of Src, cilengitide-induced permeability was only partially prevented by Src inhibition. This partial effect of CGP77675 is likely due to the fact that cilengitide-ligated and activated αVβ3 exerts a transdominant negative on β1 integrins insensitive to Src inhibition. On low matrix densities the transdominant negative effect results in decreased cell adhesion and increased cell detachment. On high-matrix densities the same effect is insufficient to detach cells, but might cause cellular retraction as observed at early time points (5–30 minutes after addition of cilengitide) in HUVEC cultured on plastic wells or partial cell detachment as observed for HUVEC cultured on filters at the end of the permeability assays. Although a role for integrins in controlling vascular permeability has been proposed before [47], [48], the molecular mechanisms involved remained elusive. Of interest, in a recent report the extracellular matrix protein βig-h3/TGFBI was shown to increase vascular permeability in a Src-dependent manner by binding to αVβ5 and causing dissociation of VE-cadherin from endothelial junctions [49]. Taken together our report extends these observations, by demonstrating cilengitide-induced increased vascular permeability of HUVEC monolayers though combined Src-dependent disruption of VE-cadherin localization at cell-cell contacts, and Src-independent cell retraction consistent with a transdominant negative effect on β1 integrins.

These observations raise a number of questions that need to be addressed in future studies. One question relates to the potential role of αVβ5 (the second integrin targeted by cilengitide) in these effects. On HUVEC, αVβ5 is likely not to play a significant role since it is expressed at much lower levels compared to αVβ3 [37] (and data not shown). *In vivo*, however, the situation might be different as αVβ5 is also highly expressed on angiogenic endothelia and its ligation was shown to promote increased vascular permeability in response to angiogenic growth factors [50]. A second question relates to the mechanism of Src activation by cilengitide-bound αVβ3, and the contribution of the α and β subunit cytoplasmic domains since both domains can bind Src [3]. Another question relates to the role that soluble high-affinity ligands binding to non matrix-ligated integrins might exert on endothelial cell functions. In this perspective, this work extends previous observations demonstrating that soluble integrins ligand can induce COX-2 mRNA and protein expression [23]. This is of particular interest, since many circulating plasma proteins are natural integrin ligands (e.g. fibronectin, vitronectin, fibrinogen) and their binding to luminal integrins (i.e. not engaged in cell-matrix adhesion) may elicit important, yet largely uncharacterized, regulatory signals. A last important question is whether the permeability-promoting effect of cilengitide observed in this study may have therapeutic implications. Cilengitide is currently in clinical development in oncology. Phase I and II clinical studies have demonstrated that it is well tolerated (no dose-limiting toxicities were observed) and provided initial evidence of activity. Phase III studies in combination with chemotherapy and radiotherapy are underway in glioblastoma multiforme [21]. Cilengitide might be a particularly well-suited drug to combine with chemotherapeutic agents with the purpose to improve chemotherapy delivery to tumors, which is one of the limiting events in cancer therapies.

## Materials and Methods

### Cells and culture media, antibodies and reagents

*Cells and culture media*: HUVEC cells were prepared and cultured as described previously [37] and were used between passage 3 and 5. *Antibodies*: anti-FAK, anti-pY^576/577^FAK, anti-Src, anti-ERK1/2 and anti-phospho ERK1/2 antibodies were from Cell Signaling Technology, Inc. (Danvers, MA); anti-pY^419^Src, anti- pY^529^Src were from Sigma Aldrich (St. Louis, MO); anti-VE-cadherin mAb was from BD Transduction Laboratories™ (Franklin Lakes, NJ), anti-pY^658^VE-cadherin and anti-pY^731^VE-cadherin antibodies were from Biosource International, Inc. (Camarillo, CA), anti-β3 clone Ab1932, anti-αVβ3 (mAb, clone LM609) and anti-total-β1 were from Chemicon (Temecula, CA, USA). The anti-β3 mAb clone AP-3 was obtained from Dr. T.J. Kunicki, the Scripps Research Institute (La Jolla, CA). The anti-β3 ligand-induced binding site mAbs were kindly provided by Dr. M. Ginsberg (LIBS-1), University of California San Diego (La Jolla, CA) or purchased from Abcam Inc. (CRC54), Cambridge (MA USA). Anti-β1 LIBS (HUTS-21) was from BD Biosciences Pharmingen (San Diego, CA), *Reagents*: BSA, fibronectin, collagen I, vitronectin and Cristal Violet were from Sigma-Aldrich, Buchs, Switzerland. Collagen I (PureCol®) was from Nutacon BV, Leimuiden, The Netherlands. The EMD121974 (the inner salt form of cyclic-(Arg-Gly-Asp-[D-Phe]-[N-Me-Val]) and EMD135981 (the inner salt form of cyclic-(Arg-[ß-Ala]-Asp-[D-Phe]-Val) cyclopeptides were synthesized by Dr A. Jonczyk, Merck KGaA, Darmstadt, Germany. The Src inhibitor CGP77675 was provided by Dr. D. Fabbro, Novartis AG (Basel, Switzerland), VEGF was purchased from R&D Systems (Abingdon, UK).

### FACS analysis

HUVEC were collected by trypsinization, washed twice in PBS/5% FCS and incubated with relevant primary antibodies for 30 minutes at 4°C. After washing in cold PBS, cells were incubated with a secondary PE-labeled antibody for 30 minutes at 4°C. Cells were washed and analyzed with a FACScan II® and Cell Quest® software (Becton Dickinson, Mountain View, California).

### Immunofluorescence microscopy

HUVEC cells were cultured subconfluent or at confluency on glass coverslips pre-coated with fibronectin (3 mg/ml) or collagen I (1 mg/ml) placed in 12 well plates in complete M199 medium. After 16 hours, cultures were stimulated with cilengitide or EMD135981 (10 µM each) or VEGF (100 ng/ml) for 20 minutes, or otherwise at the indicated times. To inhibit Src, CGP77675 (2.5 µM) was added 15 minutes before addition of cilengitide. Cultures were then immediately fixed in 4% PFA for 10 minutes at room temperature, permeabilized with 0.1% Triton X-100, blocked with 1% BSA and incubated for 1 hour with the relevant primary antibodies (5 µg/ml). After washing, cells were incubated with a Cy5 or FITC-conjugated secondary antibody. DAPI was used to counterstain nuclei. Stained cells were mounted in Prolong Antifade medium (Molecular Probes, Invitrogen) and viewed by epifluorescence microscopy (Axioplan with objective EC Plan Neofluar 40×/1.3 oil ph 3, Carl Zeiss AG, Zürich). Images were acquired with an Axiocam camera (Carl Zeiss AG) and the Axiovision program (release 4.7, Carl Zeiss AG) and processed (zooming, gamma and contrast adjustments) with Adobe Photoshop CS3 (Adobe Systems Inc. San Jose, CA)

### Quantification of VE-cadherin immunostaining

Quantification of fluorescence of VE-cadherin staining was performed with the program Metamorph 7.5 (Molecular Devices, Downingtown, PA). Briefly, a program script was defined to draw a line region along the plasma membrane of each cell on the images (magnification 40×). Then, segment regions (i.e. squares of 2.4 µm×6.4 µm - length×width) were created along the line and, in each segment region, the fluorescence was measured according to a threshold defined from a negative control. The measured fluorescence average intensities were then normalized within each segment by multiplying the fluorescence average intensity by the ratio of the threshold area divided by the total area. These normalized intensities (NI) were then arbitrarily divided in two groups: group 0 for NI<5; group 1 for 5<NI, corresponding to absent or faint *vs* intense labeling, respectively. On each image these groups were counted and plotted as shown in the Figure 6b. Quantification was performed on 30 cells per conditions.

### SDS-PAGE and Western blotting

HUVEC cells were treated with the different compounds as described in the text or figure legends. Total cell lysates (20 µl per lane) were resolved by 7.5% SDS-PAGE and blotted onto Immobilon-P membranes (Millipore, Volketswil, Switzerland). Membranes were blocked with 5% BSA prepared in 1× TBS-T. Primary antibodies were prepared as 1∶2000 dilutions in 1× TBS-T with 3% BSA and added to the membrane overnight at 4°C. Phosphoproteins were always detected first prior stripping the membranes to detect total proteins. Membranes were then washed and processed for HRP-coupled secondary antibody according to standard laboratory protocols and the ECL system was used for detection (Amersham-Pharmacia Biotech, Dübendorf, Switzerland). Membranes were reprobed for actin to assess equal loading of the samples. To compare expression levels of the different proteins, revealed Western blots were scanned and band pixelization for total and phosphorylated proteins were analyzed with the AIDA bioimaging software (raytest Isotopenmessgeraete GmbH, Straubenhardt, Germany). The number of pixels of each individual phosphoprotein bands was normalized to the corresponding total protein band pixel numbers.

### Adhesion and detachment assays

HUVEC were seeded in triplicate in 96-well plates (2×10^4^ cells/well) pre-coated with graded amounts of vitronectin (1–0.1 µg/ml), fibronectin (3–0.1 µg/ml), collagen I (1–0.01 µg/ml). After coating, the wells were saturated with 1% BSA. For short-term adhesion assays, cells were seeded in serum-free medium in the absence or presence of 10 µM cilengitide or EMD135981 and adhesion quantified after 2 hours. For survival assays, the cells were seeded in complete media and left to adhere for 16 hours followed by a 3 hours starvation in serum-free medium before addition of 10 µM cilengitide or EMD135981 for another 2 hours. To inhibit Src, CGP77675 (2.5 µM) was added 15 minutes before addition of cilengitide. At the end of the assay period, cultures were rinsed with two gentle washes with PBS (with Ca/Mg), fixed for 15 minutes with 4% paraformaldehyde and stained for 15 minutes with 0.5% Crystal Violet. Stained wells were washed and CV was extracted with 100 µl CV distain solution (29.4 g/l Na_3_-citrate in 50% ethanol) and absorbance measured at 540 nm wavelength.

### Cell viability assay

Sub-G1 DNA content assay was performed as described [51]. Briefly, HUVEC were collected as above, resuspended in 70% ice cold ethanol under vortex and incubated for 2 hours at −20°C. Cells were recovered by centrifugation and resuspended in PBS. 50 µg/ml RNase A (Roche, Basel) was added and samples were incubated at room temperature for 5 minutes before staining with propidium iodide (PI, 50 µg/ml) for 30 minutes at 37°C. Stained cells were analyzed with a FACScan II® and Cell Quest® software (Becton Dickinson, Mountain View, CA).

### *In vitro* permeability assay

The assay was adapted from [52]. Briefly, HUVEC were seeded, in triplicate, at a density of 40×10^3^ cells on polystyrene filter inserts (3 µm pore size, BD Biosciences, Basel, Switzerland, catalogue number 353096) pre-coated with fibronectin (3 µg/ml) or collagen I (1 µg/ml) or vitronectin (1 µg/ml), in 12-well plates in a total volume of 200 µl and 1 ml of complete M199 for the upper and lower chambers, respectively. After 20 hours, the medium in the upper chamber was gently exchanged with fresh one containing the paracellular permeability tracer molecule FITC-dextran [53] (av. M_r_ 40×10^3^, Sigma-Aldrich, Basel, Switzerland, catalogue number FD40S, 0.5 mg/ml final concentration) and either nothing else (control), CGP77675 (final concentration 2.5 µM), cilengitide (final concentration 10 µM) or both compounds. CGP77675 (2.5 µM) was added 15 minutes before addition of cilengitide. At given time points, 50 µl aliquots from the lower chamber were removed for measurement and replaced with 50 µl of fresh medium in order to maintain the hydrostatic equilibrium. The fluorescence of each sample diluted (1∶20) in PBS, was measured at 485/530 nm excitation/emission wavelengths. The zero time point (t = 0) was defined by diluting 50 µl (1∶20) in PBS. After the last time point, wells were fixed with 100 µl 4% PFA, stained by Crystal Violet and photographed (Axiovert 40 CFL, Carl Zeiss AG, Zürich, Switzerland).

### Statistical analysis

Results are expressed as mean±s.d.. Data were analyzed by Student's *t*-test for. *P* values<0.05 were considered significant.
